# H_1 _Antihistamines: *Current Status and Future Directions*

**DOI:** 10.1186/1939-4551-1-9-145

**Published:** 2008-09-15

**Authors:** F Estelle R Simon, Keith J Simons

**Affiliations:** 1 Department of Pediatrics and Child Health, Winnipeg, Manitoba, Canada; 2 Department of Immunology, Winnipeg, Manitoba, Canada; 3 Department of Canadian Institutes of Health Research National Training Program in Allergy and Asthma, Winnipeg, Manitoba, Canada; 4 Department of Faculty of Medicine, Winnipeg, Manitoba, Canada; 5 Department of Faculty of Pharmacy, University of Manitoba, Winnipeg, Manitoba, Canada

**Keywords:** H_1 _antihistamines, second-generation H_1 _antihistamines, nonsedating H_1 _antihistamines, allergic rhinitis, allergic conjunctivitis, urticaria, atopic dermatitis, cetirizine, desloratadine, fexofenadine, levocetirizine, loratadine

## Abstract

In this review, we compare and contrast the clinical pharmacology, efficacy, and safety of first-generation H_1 _antihistamines and second-generation H_1 _antihistamines. First-generation H_1 _antihistamines cross the blood-brain barrier, and in usual doses, they potentially cause sedation and impair cognitive function and psychomotor performance. These medications, some of which have been in use for more than 6 decades, have never been optimally investigated. Second-generation H_1 _antihistamines such as cetirizine, desloratadine, fexofenadine, levocetirizine, and loratadine cross the blood-brain barrier to a significantly smaller extent than their predecessors. The clinical pharmacology, efficacy, and safety of these medications have been extensively studied. They are therefore the H_1 _antihistamines of choice in the treatment of allergic rhinitis, allergic conjunctivitis, and urticaria. In the future, clinically advantageous H_1 _antihistamines developed with the aid of molecular techniques might be available.

## 

Histamine, a natural body constituent, is synthesized from L-histidine exclusively by histidine decarboxylase, an enzyme expressed in central nervous system (CNS) neurons, gastric mucosa parietal cells, mast cells, basophils, and other cells throughout the body. Histamine plays a major role in human health, exerting its diverse effects through 4 or more types of receptors (Table 1). Through the H_1 _receptor, histamine is involved in cell proliferation and differentiation, hematopoiesis, embryonic development, regeneration, and wound healing. It is a neurotransmitter, has anticonvulsant activity, and contributes to regulation of the sleep-waking cycle, energy and endocrine homeostasis, cognition and memory [1,2].

**Table 1:** Histamine Receptors

	H_1 _Receptor	H_2 _Receptor	H_3 _Receptor	H_4 _Receptor
Receptor expression	Nerve cells, airway and vascular smooth muscle, endothelial cells, epithelial cells, neutrophils, eosinophils, monocytes/macrophages, DC, T and B cells, hepatocytes, chondrocytes	Nerve cells, airway and vascular smooth muscle, endothelial cells, epithelial cells, neutrophils, eosinophils, monocytes, DC, T and B cells, hepatocytes, Chondrocytes	High expression in histaminergic neurons, eosinophils, DC, monocytes; low expression in peripheral tissues	High expression on bone marrow and peripheral hematopoietic cells, eosinophils, neutrophils, DC, T cells, basophils, mast cells
Histamine function, General	↑ Pruritus, pain, vasodilation, vascular permeability, hypotension; flushing, headache, tachycardia, bronchoconstriction, stimulation of airway vagal afferent nerves and cough receptors; ↓ atrioventricular node conduction time	↑ Gastric acid secretion, vascular permeability, hypotension, flushing, headache, tachycardia, chronotropic and inotropic activity, bronchodilation, mucus production (airway)	↑ Pruritus (no mast cell involvement), ↑ nasal congestion; prevent excessive bronchoconstriction	↑ Pruritus (no mast cell involvement), ↑ nasal congestion; differentiation of myeloblasts and promyelocytes
Histamine function in allergic inflammation and immune modulation	↑ Release of histamine and other mediators; ↑ cellular adhesion molecule expression and chemotaxis of eosinophils and neutrophils; ↑ antigen-presenting cell capacity, costimulatory activity on B cells; ↑ cellular immunity (Th1), ↑ autoimmunity; ↓ humoral immunity and IgE production	↑ Eosinophil and neutrophil chemotaxis;↓ IL-12 by dendritic cells; ↑ IL-10 and development of Th2 or tolerance-inducing dendritic cells; ↑ humoral immunity;↓ cellular immunity; suppresses Th2 cells and cytokines; indirect role in allergy, autoimmunity, malignancy, graft rejection	Probably involved in control of neurogenic inflammation through local neuron-mast cell feedback loops; ↑ proinflammatory activity and APC capacity	↑ Calcium flux in human eosinophils; ↑ eosinophil chemotaxis; ↑ IL-16 production (H_2 _receptor also involved)
Histamine function in the CNS	Sleep/wakefulness, food intake, thermal regulation, emotions/aggressive behavior, locomotion, memory, learning	Neuroendocrine	Presynaptic heteroreceptor; ↓ histamine, dopamine, serotonin, noradrenaline, and acetylcholine release	To be defined

APC indicates antigen-presenting cells; DC, dendritic cells; IgE, immunoglobulin E; IL, interleukin.

Adapted from Simons and Akdis [3].

Through all 4 known types of histamine receptors, histamine also plays an important role in immune modulation and in acute and chronic allergic inflammation. Through the H_1 _receptor, it increases antigen-presenting cell capacity, increases release of histamine and other mediators from mast cells and basophils, up-regulates cellular adhesion molecule expression and chemotaxis of eosinophils and neutrophils, up-regulates Th1 priming and Th1 cell proliferation and interferon-*γ *production, and down-regulates humoral immunity. Through the H_2 _receptor, it suppresses inflammatory and effector functions. Through the presynaptic H_3 _receptor on histaminergic and nonhistaminergic neurons in the central and peripheral nervous systems, it is probably involved in control of neurogenic inflammation through mast cell feedback loops. Through the H_4 _receptor, it facilitates some proinflammatory activities (Table 1) [1].

Targeted disruption of the H_1 _receptor gene in mice results in impairment of neurological functions such as memory, learning, and locomotion, and in aggressive behavior. In addition, mice that are H_1 _receptor-deficient have immunologic abnormalities, including impaired antigen-specific B-cell and T-cell responses [1-3].

All 4 types of histamine receptors are heptahelical transmembrane molecules that transduce extracellular signals, by way of G-proteins, to intracellular second messenger systems. Histamine receptors have constitutive activity, which is defined as the ability to trigger downstream events, even in the absence of ligand binding. The active and inactive states of these receptors exist in equilibrium; at rest, the inactive state isomerizes with the active state and vice versa [2,3].

### H_1 _antihistamines

H_1 _antihistamines act as inverse agonists that combine with and stabilize the inactive conformation of the H_1 _receptor, shifting the equilibrium toward the inactive state. H_1_receptor polymorphisms have been described, although it is not yet clear how they influence the clinical response to H_1 _antihistamines. Human H_1 _receptors have approximately 45% homology with muscarinic receptors [2,3].

H_1 _antihistamines down-regulate allergic inflammation through the H_1 _receptor, either directly or indirectly through nuclear factor-κB, an ubiquitous transcription factor, through which they down-regulate antigen presentation, expression of proinflammatory cytokines and cell adhesion molecules, and chemotaxis. In addition, through their effects on calcium ion channel activity, H_1 _antihistamines decrease mediator release; however, this effect is only seen at high H_1_-antihistamine concentrations [2].

Traditionally, H_1 _antihistamines have been classified into 6 chemical groups: alkylamines, ethanolamines, ethylenediamines, phenothiazines, piperazines, and piperidines. Currently, the most commonly used classification system is a functional one, in which H_1 _antihistamines are classified as either first-generation medications that readily cross the bloodbrain barrier and potentially sedate and impair cognitive and psychomotor function, or second-generation drugs that cross the blood-brain barrier to a minimal extent and are relatively nonsedating and nonimpairing[2-4] (Table 2).

**Table 2:** H_1 _Antihistamines: Chemical and Functional Classification

Functional Class		
	
Chemical Class	First Generation	Second Generation
Alkylamines	Brompheniramine, chlorpheniramine, dimethindene,**,^‡ ^pheniramine,^‡ ^triprolidine*	Acrivastine*
Piperazines	Buclizine, cyclizine, hydroxyzine,* meclizine, oxatomide**	Cetirizine,* levocetirizine*
Piperidines	Azatadine, cyproheptadine, diphenylpyraline, ketotifen^‡^	Astemizole,** bilastine,** desloratadine,* ebastine,** fexofenadine,* levocabastine,^‡ ^loratadine,* mizolastine,** olopatadine,^‡ ^rupatadine,** terfenadine*,**
Ethanolamines	Carbinoxamine, clemastine, dimenhydrinate, diphenhydramine, doxylamine, phenyltoloxamine**	--
Ethylenediamines	Antazoline, pyrilamine, tripelennamine	--
Phenothiazines	Methdilazine, promethazine	--
Other	Doxepin^§^	Azelastine,^‡ ^emedastine,^‡ ^epinastine^‡^

*Acrivastine is related tripolidine. Cetirizine is a metabolite of hydroxyzine, levocetirizine is an enantiomer of cetirizine, desloratadine is a metabolite of loratadine, and fexofenadine is a metabolite of terfenadine.

**In the United States, these H_1 _antihistamines are not yet approved, have never been approved, or have had approval withdrawn.

^‡^The H_1 _antihistamines azelastine, emedastine, epinastine, ketotifen, levocabastine, and olopatadine are available in ophthalmic formulations; and azelastine, dimethindene, levocabastine, and olopatadine are available in intranasal formulations. In some countries, azelastine, dimethindene, ketotifen, and olopatadine are also available in oral formulations.

^§^Doxepin has H_1 _and H_2 _antihistamine activities and is also classified as a tricyclic antidepressant.

Adapted from Simons [2].

H_1 _antihistamines, formerly known as H_1 _receptor antagonists or H_1 _receptor blockers, are among the most commonly used medications in the world not only for prevention and treatment of symptoms in allergic rhinitis, allergic conjunctivitis, and urticaria, in which there is good evidence for their efficacy, but also for a variety of other allergic and nonallergic diseases, in which there is no satisfactory evidence for their efficacy. More than 40 H_1 _antihistamines are available worldwide. Health care professionals and consumers generally assume that all H_1 _antihistamines approved for use are proven to be efficacious and safe. This is an incorrect assumption with regard to the first-generation medications in this class, most of which were introduced decades before clinical pharmacology studies and randomized controlled trials of medication efficacy and safety were required by regulatory agencies. In contrast, the second-generation H_1 _antihistamines, particularly cetirizine, desloratadine, fexofenadine, levocetirizine, and loratadine, have been systematically and thoroughly investigated in clinical pharmacology studies and in randomized placebo-controlled trials in allergic rhinoconjunctivitis and chronic urticaria [2-4]. In this review, we compare the clinical pharmacology, efficacy, and safety of the first-generation H_1 _antihistamines with those of the second-generation H_1 _antihistamines.

## Clinical pharmacology of H_1 _antihistamines

For most of the first-generation H_1 _antihistamines, pharmacokinetics (absorption, distribution, metabolism, and elimination) have never been optimally investigated, and pharmacodynamic studies in which drug concentrations and activity are correlated have not been performed. Clinically relevant information about the time at which maximum plasma concentrations are achieved, the terminal elimination half-life values, and the onset and duration of action is therefore available for only a few of these medications (Table 3A). Moreover, there are few prospective clinical pharmacology studies of these older H_1 _antihistamines in infants, children, the elderly, or people with impaired hepatic or renal function, and there are few studies of their interactions with other drugs, foods, or herbal products [2-5].

**Table 3A:** Pharmacokinetics and Pharmacodynamics of Oral H_1 _Antihistamines Differ in Healthy Young Adults

H_1 _Antihistamine (Metabolite)	Time to Maximum Plasma Concentration (t_max_, h) After a Single Dose	Terminal Elimination Half-life (t1/2, h)	Clinically Relevant Drug/Drug Interactions*	Onset/Duration of Action,** h
First generation				
Chlorpheniramine^‡^	2.8 ± 0.8	27.9 ± 8.7	Possible	3/24
Diphenhydramine^‡^	1.7 ± 1.0	9.2 ± 2.5	Possible	2/12
Doxepin^‡^	2	13	Possible	n/a
Hydroxyzine^‡^	2.1 ± 0.4	20.0 ± 4.1	Possible	2/24
Second generation				
Cetirizine	1.0 ± 0.5	6.5-10	Unlikely	1/≥24
Desloratadine	1-3	27	Unlikely	2/≥24
Ebastine (carebastine)	(2.6-5.7)	(10.3-19.3)	n/a	2/≥24
Fexofenadine	2.6	14.4	Unlikely	2/24
Levocetirizine	0.8 ± 0.5	7 ± 1.5	Unlikely	1/≥24
Loratadine (descarboethoxyloratadine)	1.2 ± 0.3 (1.5 ± 0.7)	7.8 ± 4.2 (24 ± 9.8)	Unlikely	2/24
Mizolastine	1.5	12.9	n/a	1/24
Rupatadine	0.75-1.0	6 (4.3-13.0)	Possible	2/24

Results are expressed as mean ± SD, unless otherwise indicated.

*Clinically relevant drug-drug interactions are unlikely with most of the second-generation H_1 _antihistamines.

**Onset/duration of action is based on wheal and flare studies.

^‡^Five or 6 decades ago when many of the first-generation H_1 _antihistamines were introduced, pharmacokinetic and pharmacodynamic studies were not required by regulatory agencies. They have subsequently been performed for some of these drugs. Empirical dosage regimens persist; for example, the manufacturers' recommended diphenhydramine dose for allergic rhinitis is 25 to 50 mg every 4 to 6 hours, and the diphenhydramine dose for insomnia is 25 to 50 mg at bedtime. The use of sustained-action formulations persists, despite the long terminal elimination half-life values identified for medications such as chlorpheniramine.

^§^Intranasal and ophthalmic H_1 _antihistamines: t_max_, t1/2, and drug-drug interactions were determined after oral administration.

^||^Intranasal and ophthalmic H_1 _antihistamine formulations: onset and duration of action is based on usual adult dose of 1 to 2 sprays in each nostril or 1 drop in each eye.

n/a indicates information not available or incomplete.

Adapted from Simons [2].

### Pharmacokinetics of second-generation H_1 _antihistamines

For most of the second-generation H_1 _antihistamines, pharmacokinetics have been well studied (Table 3A). After oral administration, peak plasma concentrations of these medications are reached in 1 to 2 hours. Terminal elimination half-life values range from about 6 hours for cetirizine, levocetirizine, and loratadine to 27 hours for desloratadine. Some of these medications such as loratadine and desloratadine are metabolized, but others are not; for example, cetirizine and levocetirizine are eliminated mostly unchanged in the urine, and fexofenadine is eliminated mostly unchanged in the feces. The pharmacokinetics of these newer H_1 _antihistamines have been studied in healthy adults, and also in infants, children, the elderly, and individuals with impaired hepatic or renal function. In drug-drug interaction studies, few clinically relevant issues have been identified, however, additional interaction studies with foods and with herbal products are needed [2-8].

### Pharmacodynamics of second-generation H_1 _antihistamines

Pharmacodynamic studies involving suppression of the response to nasal or conjunctival allergen challenge tests are helpful in determining the onset and intensity of action of H_1 _antihistamines [9]. More commonly, however, the pharmacodynamics of H_1 _antihistamines are assessed by measuring suppression of the histamine-induced wheal and flare (erythema). In randomized placebo-controlled studies using this unique model, statistically significant and clinically relevant differences among the second-generation H_1 _antihistamines have been identified with regard to onset and intensity of action, time to peak effect, and duration of effect [2-8]. Wheal and flare suppression correlates better with tissue H_1_-antihistamine concentrations than with plasma H_1_-antihistamine concentrations, and correlates best with H_1 _receptor occupancy by free unbound drug, where such data are available [2,3,5-8,10-12].

The onset of action of orally administered second-generation H_1 _antihistamines occurs from 1 hour after oral administration (for cetirizine and levocetirizine) to 2 hours (for desloratadine, fexofenadine, and loratadine; (Table 3A). Most second-generation H_1 _antihistamines have a duration of action of at least 24 hours, facilitating once-daily dosing. Tolerance to their effects during regular daily dosing does not occur. Residual effects after discontinuation of regular daily dosing last from 1 to 4 days [2,3,5-8,10,11].

### Pharmacokinetics and pharmacodynamics of intranasal and ophthalmic H_1_-antihistamine formulations

Although some systemic absorption occurs within minutes of topical and ophthalmic formulations of H_1 _antihistamines such as azelastine, emedastine, epinastine, levocabastine, and olopatadine, and is potentially associated with transient suppression of skin test reactivity, the amount of suppression is seldom clinically relevant. The elimination half-life of these medications ranges from 7 to 40 hours (Table 3B); however, they are all administered at 6-to 12-hour intervals because of washout from the nasal mucosa or conjunctivae. No dose adjustments are required in special populations [3,9,13].

**Table 3B:** Pharmacokinetics and Pharmacodynamics of H_1 _Antihistamines for Intranasal/Ophthalmic Use

H_1 _Antihistamine (Metabolite)	Time to Maximum Plasma Concentration (t_max_, h) After a Single Dose^§^	Terminal Elimination Half-Life (t1/2, h)^§^	Clinically Relevant Drug/Drug Interactions^§^	Onset/Duration of Action, h^||^
Instranasl/Ophthalmic				
Azelastine (desmethylazelastine)	5.3 ± 1.6 (20.5)	22-27.6 (54 ± 15)	No	0.5/12
Emedastine	1.4 ± 0.5	7	No	0.25/12
Epinastine	2-3	6.5	No	0.1/12
Ketotifen	2-4	20-22	No	0.25/12
Levocabastine	1-2	35-40	No	0.25/12
Olopatadine	0.5-2	7.1-9.4	No	0.25/12

Footnote symbols are explained in the legend of Table 3.

## Efficacy of H_1 _antihistamines in allergic diseases

H_1 _antihistamines prevent and relieve allergic inflammation and associated symptoms in seasonal/intermittent (perennial/persistent) allergic rhinitis, allergic conjunctivitis, and urticaria (Table 4A). Symptom relief may be incomplete because leukotrienes and other agents released from mast cells and basophils also play a role in allergic inflammation. H_1 _antihistamines are best taken on a regular basis rather than on an as-needed basis. Few of the randomized placebo-controlled clinical trials of first-generation H_1 _antihistamines that have been performed in the past 6 decades meet current standards. In contrast, the use of second-generation H_1 _antihistamines for relief of symptoms in seasonal/intermittent and perennial/persistent allergic rhinoconjunctivitis and chronic urticaria is supported by hundreds of appropriately randomized, double-masked, placebo-controlled clinical trials lasting weeks or months, in which inclusion criteria and exclusion criteria are clearly stated, an adequate number of participants is enrolled, and attrition and adherence are appropriately documented. *Second-generation H_1 _antihistamines are therefore the H_1 _antihistamines of choice in the treatment of allergic rhinitis, allergic conjunctivitis, and chronic urticaria *[2-5,13-16].

**Table 4A:** Diseases in Which Second-Generation H_1 _Antihistamines Are Drugs of First Choice Based on Randomized Controlled Trials (Grade of Recommendation = A)

Allergic rhinitis
Allergic conjunctivitis
Chronic urticaria

### Allergic rhinoconjunctivitis

In allergic rhinoconjunctivitis, second-generation H_1 _antihistamines improve quality of life by preventing and relieving the sneezing, nasal and conjunctival itching, rhinorrhea, tearing, and conjunctival erythema of the early response to allergen. A small beneficial effect is also reported for the nasal congestion that characterizes the late allergic response. Cetirizine, desloratadine, fexofenadine, levocetirizine, loratadine, and other second-generation H_1 _antihistamines have significantly greater efficacy than placebo, as documented in well-designed, randomized, placebo-controlled trials (Table 4A). In the few published studies in which their efficacy relative to each other or to first-generation H_1 _antihistamines has been investigated, no overall superior efficacy of one H_1 _antihistamine over another has been consistently documented. Additional comparative, randomized, controlled trials of second-generation H_1 _antihistamines are needed [2-5,7,8,13-22].

Intranasal or ophthalmic H_1_-antihistamine formulations have a more rapid onset of action than oral H_1_-antihistamine formulations; for example, 15 minutes for intranasal azelastine versus 150 minutes for oral desloratadine; however, as noted previously, these formulations require administration several times daily [9,13,22].

In many individuals with allergic rhinoconjunctivitis in whom eye symptoms predominate, H_1 _antihistamines applied to the conjunctivae are the medications of choice not only for their antihistaminic effects, but also for their mast cell-stabilizing effects, and their rapid onset of action (range, 3-15 minutes). H_1 _antihistamines, whether administered orally or applied directly to the conjunctivae, have a more favorable therapeutic index than any of the other classes of medications used for allergic conjunctivitis (Table 4A) [3,13,22].

Selection of an H_1 _antihistamine for an individual with allergic rhinoconjunctivitis should be based on his/her preference for a particular H_1_-antihistamine formulation, route of administration, or dose regimen, and on considerations of potential benefits versus potential adverse effects.

Second-generation H_1 _antihistamines have similar efficacy to intranasal cromolyn, intranasal nedocromil, and leukotriene modifiers in seasonal allergic rhinitis. The combination of desloratadine or levocetirizine with montelukast might be more efficacious than monotherapy with any one of these agents; however, a combined loratadine/montelukast formulation has failed to gain US Food and Drug Administration (FDA) approval. To provide increased relief of nasal congestion, H_1 _antihistamines are sometimes marketed in fixed-dose combinations with pseudoephedrine or other decongestants. H_1 _antihistamines are less efficacious than intranasal glucocorticoids, especially for relief of nasal congestion [2,3,14,16].

### Urticaria

H_1 _antihistamines are efficacious in acute urticaria, defined as hives lasting less than 6 weeks, and in chronic urticaria, defined as hives lasting 6 weeks or more, including physical urticarias such as cholinergic, cold, aquagenic, and delayed pressure-induced urticaria. They decrease itching, reduce the number, size, and duration of wheals and flares (erythema), and improve quality of life significantly [2,3,5,23-29]. They are not efficacious in urticarial vasculitis.

In acute urticaria, both first-and second-generation H_1 _antihistamines are widely used, however, there are surprisingly few randomized controlled trials in support of this practice. In 2 different large, randomized, double-masked, placebo-controlled studies in young atopic children in which efficacy in preventing and treating acute urticaria was a planned secondary outcome, cetirizine and levocetirizine had statistically significant and clinically relevant beneficial effects [23-26].

The first-generation H_1 _antihistamines remain in widespread use for chronic urticaria, despite lack of randomized placebo-controlled efficacy trials that meet current standards, and despite concerns about their potential adverse effects. In contrast, the second-generation H_1 _antihistamines cetirizine, desloratadine, fexofenadine, levocetirizine, loratadine, and others have been well studied in chronic urticaria and are therefore the cornerstone of treatment in this disease (Table 4A) [2,3,5,7,8,27-29].

In chronic urticaria that is unresponsive to a second-generation H_1 _antihistamine in a standard dose, a variety of therapeutic strategies are recommended [26]. High (off-label) doses of second-generation H_1 _antihistamines have been prospectively tested in a few randomized, double-masked, placebo-controlled trials and may offer some advantage. Use of 2 different second-generation H_1 _antihistamines on the same day or use of a nonsedating H_1 _antihistamine in the morning and a sedating H_1 _antihistamine at night is commonly recommended based on tradition and clinical experience. Prospective, randomized, controlled trials of these treatment regimens are long overdue.

Some but not all individuals with severe chronic urticaria that is unresponsive to H_1 _antihistamines will respond to montelukast or to an H_2 _antihistamine such as cimetidine. Individuals with intractable pruritus might require a course of treatment with an immunomodulator such as an oral corticosteroid, cyclosporin, hydroxychloroquine, omalizumab, dapsone, colchicine, sulfasalazine, mycophenolate, or oral tacrolimus.

Montelukast has been studied in large, randomized, placebo-controlled trials in chronic urticaria and cyclosporine, hydroxychloroquine, and omalizumab have been studied in small, randomized, placebo-controlled trials. None of the other pharmacological interventions used in chronic urticaria refractory to antihistamine treatment have been studied in randomized, placebo-controlled trials. With the exception of antihistamines and montelukast, immunomodulators used in chronic urticaria have potentially severe adverse effects, and individuals taking them need to be monitored on a regular basis [13,23,26].

## Diseases in which H1 antihistamines are used but are not drugs of first choice

H_1 _antihistamines are administered in many diseases in which their use is not adequately supported by randomized controlled trials (Tables 4B-E).

**Table 4B:** Diseases in Which H_1 _Antihistamines Are NOT Drugs of First Choice Based on Paucity of Evidence from Randomized Controlled Trials and on Availability of More Effective Alternatives*

Atopic dermatitis
Asthma
Anaphylaxis
Nonallergic (hereditary or acquired) angioedema

*Most randomized controlled trials of H_1 _antihistamines in atopic dermatitis have not shown any significant benefit. H_1 _antihistamines do no harm in asthma and might be useful in individuals with mild seasonal allergic asthma and concomitant allergic conjunctivitis. H_1 _antihistamines relieve itching and hives in anaphylaxis but are not drugs of choice in this disease and may cause harm if their use delays epinephrine (adrenaline) treatment. In nonallergic (hereditary or acquired) angioedema, H_1 _antihistamines are not effective, and this may actually help point toward the correct diagnosis.

**Table 4C:** Diseases in Which H_1 _Antihistamines Are Used But Are Not Drugs of Choice Based on Lack of Evidence From Randomized Controlled Trials

Upper respiratory tract infection
Nonspecific cough*
Otitis media (acute otitis media, or otitis media with effusion)
Sinusitis
Nasal polyps

*Especially in children.

**Table 4D:** CNS Diseases/Clinical Situations in Which First-Generation H_1 _Antihistamines Are Used*

Insomnia
Perioperative sedation
Antiemetic effect
Analgesia
Akathisia
Serotonin syndrome
Anxiety
Migraine headache

*Safer alternatives are preferred.

**Table 4E:** Vestibular Disorders in Which First-Generation H_1 _Antihistamines Are Used*

Vertigo
Motion sickness

*Safer alternatives are preferred.

### Atopic dermatitis and other skin disorders

The evidence that H_1 _antihistamines relieve itch in atopic dermatitis is limited to a study of fexofenadine in which itching was the only outcome measure and a study of cetirizine in which off-label doses as high as 40 mg were administered. In atopic dermatitis, histamine may act as a pruritogen not only through H_1 _receptors, but also through H_3 _and H_4 _receptors; in addition, cytokines such as interleukin-31 and other agents may be important pruritogens (Table 4B) [30-32].

The use of H_1 _antihistamines to relieve symptoms in individuals with mastocytosis or to prevent and relieve itchy local allergic reactions to mosquito bites is supported by small, randomized, controlled trials [3].

### Asthma

Pretreatment with an H_1 _antihistamine provides significant protection against bronchospasm induced by histamine, adenosine-5 monophosphate, or allergen, but less protection against bronchospasm induced by exercise or other stimuli. H_1 _antihistamines decrease symptoms significantly in many individuals with concurrent seasonal allergic rhinitis and mild asthma; however, they have a greater effect on the rhinitis symptoms than on the asthma symptoms. In an 18-month-long study in very young children with atopic dermatitis and house-dust mite or grass sensitization who were at risk for developing asthma, cetirizine treatment delayed asthma onset, but in a subsequent study in highly atopic young children, this observation was not confirmed with levocetirizine (Table 4B) [2,3,5,33,34].

### Anaphylaxis

A recent Cochrane collaboration review of 2070 studies of H_1 _antihistamines in anaphylaxis did not reveal any study that provided evidence for the use of H_1 _antihistamines in this disease. Individuals who require first-aid treatment of anaphylaxis occurring in a community setting should not depend on an oral H_1 _antihistamine because onset of action takes 1 to 2 hours, and although these medications decrease itch and hives, they do not relieve upper or lower respiratory tract obstruction or circulatory collapse and do not prevent fatality (Table 4B) [35].

### Nonallergic angioedema

Nonallergic angioedema *without *associated itching or urticaria may be hereditary (types I, II, and III) or acquired--for example, associated with the use of an angiotensinconverting enzyme inhibitor or with malignancy. In an individual with angioedema who has no associated itching or hives, *lack of response to H_1 _antihistamine treatment *points to the need for appropriate investigations for nonallergic (hereditary or acquired) angioedema (Table 4B) [13].

### Other

H_1 _antihistamines are widely used to relieve symptoms of upper respiratory tract infections, nonspecific cough, acute otitis media, otitis media with effusion, sinusitis, and nasal polyps; however, the published evidence does not support their use in these disorders (Table 4C) [36-39].

### Central nervous system and vestibular system disorders: the unfavorable therapeutic index of first-generation H_1 _antihistamines

Diphenhydramine, doxylamine, and pyrilamine are the most widely used sleep-inducing medications in the world (Table 4D). They are not, however, medications of choice for insomnia because they distort sleep architecture (as evidenced by a decrease in rapid eye movement sleep), increase rebound wakefulness, and potentially cause other adverse effects (Table 5). H_1 _antihistamines are also still used for treatment of akathisia, serotonin syndrome, anxiety, migraine, and other CNS disorders. Diphenhydramine, hydroxyzine, cyproheptadine, and promethazine are still administered for perioperative sedation and for analgesia; however, there are serious concerns about their potential adverse effects in these settings. Indeed, promethazine has received a black box warning from the US FDA regarding its use in young children because of its association with CNS adverse effects, respiratory depression, and death in this age group [2,3].

For antiemetic effects and for prevention and treatment of motion sickness, vertigo, and related disorders, the first-generation H_1 _antihistamines dimenhydrinate, diphenhydramine, meclizine, and promethazine are used to block the histaminergic signal from the vestibular nucleus to the vomiting center in the medulla. These medications have an unfavorable benefit-to-risk ratio, and because of CNS adverse effects, military pilots and commercial airline pilots are prohibited from using them. Second-generation H_1 _antihistamines do not prevent motion sickness (Table 4E) [2,3].

## Adverse effects of H_1 _antihistamines

### First-generation H_1 _antihistamines

First-generation H_1 _antihistamines potentially cause a wide variety of adverse effects in many body systems (Table 5) [2-4,20,40]. The main concern, however, is that all first-generation H_1 _antihistamines, even when administered in manufacturers' recommended doses, have the proclivity to interfere with neurotransmission by histamine at CNS H_1 _receptors. This potentially leads to adverse CNS symptoms such as drowsiness, sedation, somnolence, fatigue, and headache. More importantly, it potentially impairs cognitive function, memory, and psychomotor performance. Positron emission tomography with [11]C-doxepin as the positron-emitting ligand reveals that these medications occupy more than 70% of the CNS H_1 _receptors [41]. Blood-brain barrier penetration is related to their lipophilicity, relatively low molecular weights, and lack of substrate recognition by the P-glycoprotein efflux pump expressed on the luminal surfaces of nonfenestrated endothelial cells in the CNS vasculature. Central nervous system penetration is also documented in randomized controlled studies involving electroencephalographic monitoring, sleep latency measurements, and standardized performance tests ranging from simple reaction time tests to complex sensorimotor tasks, for example, computer-monitored driving [2-4,10,42,43].

**Table 5:** Adverse Effects of First-Generation H_1 _Antihistamines Versus Second-Generation H_1 _Antihistamines

	First Generation*,**,^‡^	Second Generation**,^‡^
CNS (mechanism: interference with neurotransmitter effect of histamine through H_1 _receptor)	After usual doses, may cause drowsiness, fatigue, somnolence, dizziness, impairment of cognitive function, memory, and psychomotor performance, headache, dystonia, dyskinesia, agitation, confusion, and hallucinations.	None with fexofenadine at doses up to 360 mg (off label); none with desloratadine 5 mg or loratadine 10 mg, although dose-related CNS effects may occur at higher doses; cetirizine doses 10 mg or higher may cause sedation in adults.
	May cause adverse CNS effects in newborns if taken by the mother immediately before parturition; may cause irritability, drowsiness, or respiratory depression in nursing infants	No CNS adverse effects reported in newborns or nursing infants
Cardiac^§ ^(mechanisms: multiple; antimuscarinic effects; α-adrenergic receptor blockade; blockade of cardiac ion currents [I_Kr _and, less commonly, I_Na_, I_to_, I_Ki_, and I_Ks_])	Dose-related sinus tachycardia; reflex tachycardia, prolonged atrial refractive period, and supraventricular arrhythmias; dose-related prolongation of the QTc interval and ventricular arrhythmias reported for cyproheptadine, diphenhydramine, doxepin, hydroxyzine, promethazine, and others	No major concerns in any country (such as the United States or Canada), in which regulatory approval was withdrawn for astemizole and terfenadine
Other sites (mechanisms: blockade of muscarinic, α-adrenergic, and serotonin receptors)	After usual doses: may cause mydriasis (pupillary dilation), dry eyes, dry mouth, urinary retention and hesitancy, decreased gastrointestinal motility, constipation, memory deficits; peripheral vasodilation, postural hypotension, dizziness; appetite stimulation and weight gain (cyproheptadine, ketotifen); contraindicated in individuals with glaucoma or prostatic hypertrophy	None reported
Toxicity after overdose (mechanisms: multiple)	CNS effects such as extreme drowsiness, lethargy, confusion, delirium, and coma in adults; paradoxical excitation, irritability, hyperactivity, insomnia, hallucinations, seizures, and respiratory depression/arrest in infants and young children; in both adults and children, CNS adverse effects predominate over cardiac adverse effects; death may occur within hours after ingestion of drug in untreated patients; rhabdomyolysis has also been reported	No serious toxicity or fatality reported
Abuse of drugs (mechanisms: through H_1 _and other receptors in the CNS)	Euphoria, hallucinations and "getting high" reported for diphenhydramine, dimenhydrinate, and others	None reported
Teratogenicity after use in pregnancy^||^	FDA Category B (chlorpheniramine, diphenhydramine) or C (hydroxyzine, ketotifen)	FDA Category B (cetirizine, emedastine, levocetrizine, loratadine) or C (azelastine, epinastine, desloratadine, fexofenadine, olopatadine)
Carcinogenicity/tumor promotion	None reported in humans	None documented in humans

*Most first-generation H_1 _antihistamines have not been prospectively studied for their adverse effects. The information is based on descriptions of adverse effects in case reports and case series published during the past 60 to 70 years. First-generation H_1 _antihistamines, particularly in the phenothiazine class, have been associated with sudden infant death syndrome, although causality has never been proven. First-generation H_1 _antihistamines such asdiphenhydramine or doxepin, applied topically to the skin, may cause contact dermatitis and if applied to abraded skin, they potentially cause systemic adverse effects.

**Rarely, bothfirst-and second-generation H_1 _antihistamines are reported to cause adverse effects for which the mechanisms are incompletely understood:fixed-drug eruption, photosensitivity, urticaria, anaphylaxis, fever, liver enzyme elevation/hepatitis, agranulocytosis.

^‡^Intranasal or ophthalmic formulations of H_1 _antihistamines such as azelastine, emedastine, epinastine, levocabastine, ketotifen, and olopatadine have not been optimally studied using objective tests for adverse effects. They maycause stinging or burning upon application. Some of these formulations contain benzalkonium chloride 0.01%, which can dissolve contact lenses and should therefore be applied 10 minutes before insertion of lenses. Azelastine and emedastine may cause dysgeusia (bitter taste).

^§^I_Kr_, rapid component of the delayed rectifier potassium current; I_Na_, sodium current; I_to_, transient outward potassium current; I_Ki_, inward rectifying current; I_Ks_, slow component of the delayed rectifier potassium current.

^||^US FDA.

Category A, animal studies and human studies negative; no H_1 _antihistamines in this category.

Category B, animal studies negative, human data not available; or animal studies positive, human data negative.

Category C, animal studies positive, human data not available; or neither animal nor human data available.

Category D, animal studies positive or negative; human studies or reports positive.

Adapted from Simons [2].

Impairment of CNS function by first-generation H_1 _antihistamines in usual doses has been documented in the absence of CNS symptoms. Tolerance to adverse CNS effects does not necessarily occur. After taking one of these older medications at bedtime, some individuals have residual CNS adverse effects the next morning, the so-called antihistamine hangover. The CNS effects of a first-generation H_1 _antihistamine are similar to, and exacerbate, those produced by ethanol or by other CNS-active chemicals [2,3]. Prospective, long-term, randomized, controlled studies of the safety of these older H_1 _antihistamines have never been published.

### Second-generation H_1 _antihistamines

In contrast, the newer H_1 _antihistamines penetrate poorly into the CNS and occupy from 0% (fexofenadine, in doses up to 360 mg) to 30% (cetirizine, in above-label doses of 20 mg) of H_1 _receptors in the CNS, as documented by positron emission tomographic scan studies. The results of these studies correlate well with electroencephalographic monitoring, including sleep latency tests, and with standardized performance tests. The second-generation H_1 _antihistamines therefore have a low likelihood of causing CNS effects, although some of them, such as cetirizine and loratadine, potentially cause sedation when manufacturers' recommended doses are exceeded. Fexofenadine, in off-label doses up to and including 360 mg daily, is the least sedating of these medications and is therefore considered to be the H_1 _antihistamine of choice for airline pilots and people in other safety-critica[2-4,10,42,43]

The second-generation H_1 _antihistamines do not exacerbate the CNS effects of coadministered alcohol or other CNS-active substances. In real-world prescription-event monitoring studies conducted in thousands of individuals with allergic rhinitis during the first 30 days after introduction of a new H_1 _antihistamine in the United Kingdom, a low risk of sedation was reported for cetirizine, desloratadine, fexofenadine, levocetirizine, and loratadine [44,45].

The potential cardiac toxicity of H_1 _antihistamines that occurs because of blockade of cardiac ion currents, most commonly the I_Kr _current, is not an H_1 _antihistamine class effect. Since withdrawal of regulatory approval for astemizole and terfenadine almost 2 decades ago, the second-generation H_1 _antihistamines remaining in use are free from potential cardiac adverse effects (Table 5) [2-4].

Randomized, controlled trials documenting the long-term safety of these medications have been published. These include randomized, controlled, masked studies of 6 to 12 months' duration with desloratadine, fexofenadine, and levocetirizine in adults, and of 12 to 18 months' duration with cetirizine, levocetirizine, and loratadine in very young children [20,21,46-48].

### H_1_-antihistamine overdoses

After overdose with a first-generation H_1 _antihistamine, CNS symptoms predominate (Table 5). In adults, these symptoms usually culminate in extreme drowsiness, confusion, and coma. In infants and children, paradoxical CNS excitation, with symptoms of irritability, hyperactivity, insomnia, hallucinations, and seizures may occur. Some first-generation H_1 _antihistamines also potentially cause dose-related cardiac adverse effects, including sinus tachycardia, reflex tachycardia, supraventricular arrhythmias, and after intentional large overdose, for example, diphenhydramine 0.5 to 1 g, prolongation of the QT interval with ventricular arrhythmias and torsade de pointes has been documented [2-5].

Deaths attributed to first-generation H_1 _antihistamines caused by accidental overdose, suicide, and homicide (of infants) have been reported in the literature for more than 6 decades [40]. Diphenhydramine overdoses are so frequently reported to poison control centers in the United States that evidence-based guidelines have been published to facilitate their management [49].

Massive (eg, up to 20- to 30-fold) overdoses of second-generation H_1 _antihistamines such as cetirizine, fexofenadine, and loratadine have not been causally linked with serious CNS or cardiovascular adverse events or deaths (Table 5) [2,3,34].

### Use of H_1 _antihistamines in the elderly

Elderly people have increased vulnerability to adverse effects from any CNS-active chemical. Widespread use of first-generation H_1 _antihistamines not only for allergic rhinoconjunctivitis and urticaria, but also for treatment of insomnia and other clinical problems is a particular concern because of their potential to cross the blood-brain barrier, impair neurotransmission at CNS H_1 _receptors, and cause adverse CNS effects such as drowsiness, confusion, and agitation.

Polymedication is common in the elderly and the potential for first-generation H_1 _antihistamines to interact with other drugs or herbal products is therefore increased in this age group. Potential antimuscarinic effects such as mydriasis, dry eyes and dry mouth, urinary retention, urinary hesitancy, and constipation, and potential anti-*α*-adrenergic effects such as dizziness and hypotension from first-generation H_1 _antihistamines are also a concern (Table 5) [2-4,20].

### Use of H_1 _antihistamines in pregnancy and lactation

Regulatory agencies such as the US FDA and the European Medicines Agency scrutinize all medications carefully for potential teratogenicity. No H_1 _antihistamines have been designated as FDA Category A, denoting negative studies in animals and negative human data. A few H_1 _antihistamines, including chlorpheniramine, diphenhydramine, cetirizine, levocetirizine, loratadine, and the ophthalmic formulation of emedastine, have been designated as FDA Category B. This denotes that either studies in animals have shown no adverse effects and data in humans are not available, or studies in animals have shown adverse effects but studies in humans have not shown these effects. These medications are therefore considered to be relatively safe for use if needed in pregnancy. Other H_1 _antihistamines are designated as Category C. This denotes that either animal studies are positive and human data are not available, or neither animal nor human data are available. H_1 _antihistamines that are not approved for use in the United States, for example, ebastine, mizolastine, and rupatadine are not categorized by the FDA (Table 5) [2,3].

H_1 _antihistamines are secreted into breast milk. Nursing infants receive approximately 0.1% of an orally administered maternal dose. First-generation H_1 _antihistamines have been reported to cause sedation and other adverse effects in these infants (Table 5) [2,3].

### Use of H_1 _antihistamines in infants and young children

First-generation H_1 _antihistamines are widely used in infants and young children not only for allergic rhinoconjunctivitis and urticaria, but also for colds, cough, and other ailments, and for insomnia relief. Because of lack of efficacy data and concerns about safety, manufacturers in the United States and some other countries are being urged to voluntarily recall over-the-counter cold and cough preparations for children younger than 2 years and to add the warning, "Do not use to sedate children," to the label of first-generation H_1_-antihistamine preparations [5,50].

The second-generation H_1 _antihistamines cetirizine, fexofenadine, and desloratadine have been prospectively studied in infants aged 6 to 11 months in placebo-controlled trials lasting a few weeks [3]. The long-term safety profiles of cetirizine, levocetirizine, and loratadine are similar to placebo, as confirmed in randomized, masked, controlled trials in young children aged 12 to 36 months. Studies of all 3 medications involved monitoring of adverse event reports, body mass and height measurements, blood hematology and chemistry tests, and for some of them, electrocardiograms, monitoring of developmental milestones and behavior, and objective tests of intellectual performance [46-48].

## Summary and future directions

The molecular basis for H_1 _antihistamine action as inverse agonists rather than as antagonists or blockers has been briefly reviewed. The first-generation potentially sedating H_1 _antihistamines, none of which have ever been optimally investigated in humans, have been described briefly. The second-generation nonsedating H_1 _antihistamines, most of which are well investigated and are the H_1 _antihistamines of choice for treatment of allergic rhinitis, allergic conjunctivitis, and chronic urticaria, have been discussed more extensively. In contrast to the first-generation H_1 _antihistamines, the second-generation medications in the class are relatively free from adverse effects, including CNS and cardiac toxicity, when administered in standard doses and even if taken in overdose.

Most of the second-generation H_1 _antihistamines currently in use have been identified by screening and structural modification of preexisting medications in the class. For example, cetirizine is a metabolite of hydroxyzine, levocetirizine is the active R-enantiomer of cetirizine, desloratadine is a metabolite of loratadine, and fexofenadine is a metabolite of terfenadine. New H_1 _antihistamines continue to be developed and introduced for clinical use[51,52]; however, such medications should be scrutinized closely because they may or may not represent important clinically relevant advances when compared with existing second-generation medications in the class. To date, no second-generation H_1 _antihistamine appears to have superior overall efficacy to the others, although some are safer than others [2-4].

The terms third generation, new generation, or next generation have been used to market some new H_1 _antihistamines; however, this designation should be reserved for clinically advantageous H_1 _antihistamines designed with the use of molecular techniques that might be available in the future [4]. Some of these medications might also have the intrinsic ability to down-regulate histamine at H_2_, H_3_, or H_4 _receptors or to down-regulate leukotrienes or cytokines [2-4,32,53].
